# Efficacy of commercialised extracorporeal shock wave lithotripsy service: a review of 589 renal stones

**DOI:** 10.1186/s12894-017-0249-8

**Published:** 2017-07-27

**Authors:** Tommy Kjærgaard Nielsen, Jørgen Bjerggaard Jensen

**Affiliations:** Department of Urology, Hospitalsenheden Vest, Holstebro, Denmark

**Keywords:** Urinary calculi, Eswl, Lithotripsy, Mobile, Shockwave, Stones, Ureteral stent

## Abstract

**Background:**

Extracorporeal shockwave lithotripsy (ESWL) is the management of choice for renal stones 20 mm or smaller, with a stone clearance rate of up to 89%. The purpose of the present is to investigate the efficacy of a commercialised ESWL service, being performed as an outsourced treatment using a mobile lithotripsy system on an outpatient basis. Furthermore, the study aims to evaluate the risk of needing treatment with an internal ureteral double-J stent (JJ) after ESWL treatment.

**Methods:**

During an eight-year period, 461 patients with a total of 589 renal stones were treated using a mobile lithotripsy system at a single Danish institution. A commercial company performed all treatments using a Storz Modulith SLK® system. Each stone was prospectively registered according to size, intra renal location and the presence of a JJ at the time of treatment. The number of required ESWL treatments and auxiliary procedures were retrospectively evaluated.

**Results:**

The success rate after the initial ESWL procedure was 69%, which increased to an overall success rate of 93% after repeated treatment. A negative correlation was found between stone size and the overall success rate (*r* = −0.2, *p* < 0.01). The upper calyx was associated with a significantly better success rate, but otherwise intra renal stone location was not predictive for treatment success. A total of 17 patients (2.9%) required treatment with a JJ after the ESWL procedure. No significant difference was observed between the stone size or intra renal location and the risk of needing treatment with JJ after ESWL.

**Conclusions:**

Commercialised ESWL treatment can achieve an overall success rate of more than 90% using a mobile lithotripsy system. As expected, an inverse relation between stone size and success rate was found. Patients who do not require treatment with a JJ prior to ESWL will only rarely need treatment with a JJ after ESWL, irrespective of stone size and intra renal stone location.

## Background

Extracorporeal shock wave lithotripsy (ESWL) was introduced in the 1980s and is still considered an effective and minimal invasive treatment of symptomatic as well as asymptomatic nephrolithiasis.

In most cases, treatment can be preformed on an outpatient basis with none or minimal anaesthesia. ESWL is a well-established management for nephrolithiasis and is the suggested first line treatment together with retrograde intrarenal surgery (RIRS) for stones smaller than 2 cm in the renal pelvis or upper/middle calyx, according to European Association of Urology (EAU) guidelines. However, it is still debated what the best practice is for patients with lower pole stones. The overall efficacy of ESWL for nephrolithiasis depends mainly on stone size, location, stone composition, patient habitus and performance of ESWL [1, 2]. Reports from high volume centres with static machines suggests stone clearance rates of 86–89%, 71–83%, 73–84% and 37–68% for stones in the renal pelvis, upper calyx, middle calyx and lower pole calyx, respectively [3–6].

Outsourcing of EWSL procedures has routinely been used in Denmark where a static system was unavailable. Lithotripsy services were performed by dedicated technicians who visited hospitals periodically and provided treatments using a mobile lithotripsy system. However, such outsourcing of medical procedures may lack clinical ownership and inconsistencies, thus potentially risking inferior clinical results [7]. There are only a very limited number of studies that explores the efficacy of mobile lithotripter services being performed by commercial companies. And as health care services continuously are being outsourced to private operators it seems reasonable to investigate the efficacy of such treatment.

This study presents an assessment of the efficacy of such commercialised lithotripsy service in terms of stone free rate and auxiliary procedures. Furthermore, the study assesses the risk of having treatment with a JJ after the ESWL procedure due to complications associated with acute ureteral obstruction.

## Methods

During an eight-year period a total of 461 patients (261 males and 200 females) with a mean age of 59 years (range 20–90 years) and with a total of 589 renal stones, underwent commercialised ESWL treatment at The Regional Hospital Holstebro, Denmark. All patients were prior to treatment radiographically diagnosed by non-contast computed tomography (CT).

Information regarding stone size (<5 mm, 5–10 mm, 10-20 mm and >20 mm) and intra renal stone location (upper calyx, middle calyx, lower calyx and renal pelvis), as well as treatment with JJ prior to ESWL was registered at the time of treatment. Treatment outcome and auxiliary procedures were retrospectively evaluated. Stone characteristics are summarized in Table 1.

**Table 1:** Distribution of intra renal stone location and stone size. Number of stones (%)

	Total	< 5 mm	5–10 mm	10–20 mm	>20 mm
Upper calyx, n (%)	77 (13)	23 (30)	46 (60)	8 (10)	0
Middle calyx, n (%)	76 (13)	21 (28)	47 (62)	8 (10)	0
Lower calyx, n (%)	257 (44)	46 (18)	147 (57)	59 (23)	5 (2)
Renal pelvis, n (%)	179 (30)	15 (9)	101 (56)	56 (31)	7 (4)

All treatments were preformed on an outpatient basis by a commercial company (MLS Medical, Denmark) using a mobile lithotriptor system (Storz Modulith® SLK, Stortz Medical, Switzerland) featuring X-ray and ultrasound localization. Experienced technicians performed all treatment and were assisted by the responsible urologist. Treatment protocols followed producer recommendations: a) maximum 3500 impulses by 0,82 mJ/mm2 and later b) maximum 4000 impulses by 0,77 mJ/mm2. The amount of analgesia used was individualised and was at the discretion of the treating urologist. Post-ESWL stone-expulsive treatment such as tamsulosin was not used.

Stone fragmentation was assessed two weeks after treatment using non-contrast CT and patients were considered stone free if CT confirmed stone clearance or the persistence of fragments smaller than 2 mm in maximum diameter. The treating policy towards ESWL was liberal, and there was no maximum fixed number of ESWL attempts as long as progress was observed. If there was no progress after two ESWL attempts auxiliary procedures was initiated.

The department had a restrictive policy towards the use of JJ in patients with nephrolithiasis. Thus, patients were not routinely treated with a JJ either prior to or after ESWL as a result of stone size or intra renal stone location. A JJ was only placed if the patient had hydronephrosis or was discomforted in such a degree that it could not be controlled with oral analgesics.

For statistical analysis of the data, chi-squared, Fischer’s exact or Student’s t-test were used as appropriate. In correlation analysis Pearson correlation coefficient were estimated. Statistical significance was evaluated based on a two-sided significance level of 0.05. Data analysis was performed using STATA v.14 software (StataCorp, LP, USA).

## Results

A total of 408 stones (69%) were successfully treated with the initial ESWL procedure, which increased to 549 stones (93%) after repeated ESWL treatments (average number of treatments per stone = 1.4). A total of 40 stones (7%) did not respond to ESWL treatment and were treated with RIRS (*n* = 26) or percutaneous nephrolithotomy (PNL) (*n* = 14).

The mean number of ESWL sessions required for stones <5 mm, 5-10 mm, 10-20 mm and >20 mm were 1.1, 1.3, 1.6 and 1.4, respectively (*p* < 0.01). The overall ESWL success rates were 98%, 95%, 88%, and 75% respectively (*p* < 0.05). A correlation analysis between stone size and the overall ESWL success rate demonstrated a significant decrease in success rate as stone size increases (*r* = −0.2, *p* < 0.01). Treatment outcomes according to stone size are summarized in Table 2.

**Table 2:** Treatment outcome in relation to stone size

	< 5 mm *n* = 105 (18%)	5–10 mm *n* = 341 (58%)	10–20 mm *n* = 131 (22%)	> 20 mm *n* = 12 (2%)
Mean no of sessions (range)	1.1 (1–3)	1.3 (1–4)	1.6 (1–4)	1.4 (1–3)
Success after initial ESWL, n (%)	90 (86)	245 (72)	67 (51)	6 (50)
Accumulated success after 2nd	102 (97)	303 (89)	99 (76)	8 (67)
ESWL, n (%)				
Accumulated success after ≥3rd	103 (98)	322 (95)	115 (88)	9 (75)
ESWL, n (%)				
JJ present at ESWL, n (%)	5 (5)	29 (9)	20 (15)	2 (17)
Acute JJ placement after ESWL, n (%)	0	11 (3)	6 (5)	0

The mean number of ESWL sessions for stones in upper-, middle-, lower calyx and renal pelvis was 1.3, 1.2, 1.4 and 1.3 respectively (*p* > 0.05). The overall ESWL success rates were 99%, 95%, 93% and 91% respectively. With the exception of the upper calyx (*p* < 0.05), intra renal stone location did not prove to be predictive for ESWL efficacy (*p* > 0.05). Treatment outcomes according to intra renal stone location are summarized in Table 3.

**Table 3:** Treatment outcome according to intra renal stone location

	Upper Calyx *n* = 77 (13%)	Middle Calyx *n* = 76 (13%)	Lower Calyx *n* = 257 (44%)	Renal Pelvis *n* = 179 (30%)
Mean no of sessions (range)	1.3 (1–3)	1.2 (1–3)	1.4 (1–4)	1.3 (1–4)
Success after 1st ESWL, n (%)	53 (69)	59 (78)	171 (67)	124 (69)
Accumulated success after 2nd ESWL, n (%)	73 (95)	69 (91)	215 (84)	154 (86)
Accumulated success after ≥3rd ESWL, n (%)	76 (99)	72 (95)	238 (93)	163 (91)
JJ present at ESWL, n (%)	3 (4)	3 (4)	17 (7)	33 (18)
JJ placement after ESWL, n (%)	0	1 (1)	10 (4)	6 (3)

The majority of patients (90.5%) did not have a JJ at the time of ESWL treatment. As expected, significantly more patients were treated with a JJ prior to ESWL when stones were located in the renal pelvis compared to other intra renal stone locations (*p* = 0.05). Table 4 provides the efficacy rates according the presence of a JJ. A total of 17 patients (2.9%) were treated with a JJ due to post-ESWL hydronephrosis, pain or steinstrasse. The median time from ESWL to treatment with a JJ were 31 days (95%CI±13). No significant difference was observed between either stone size or intra renal stone location and the risk of needing treatment with a JJ after ESWL (*p* > 0.05).

**Table 4:** Effect of JJ on stone clearance. Number of stones (%)

	Lower Calyx, *n* = 257		Renal Pelvis, *n* = 179		Stones 5–10 mm, *n* = 341		Stones 10–20 mm, *n* = 131	
	JJ not present *n* = 240	JJ present *n* = 17	JJ not present *n* = 146	JJ present *n* = 33	JJ not present *n* = 312	JJ present *n* = 29	JJ not present *n* = 111	JJ present *n* = 20
Succes after 1st ESWL	163 (68)	8 (47)	106 (73)	18 (55)	226 (72)	19 (66)	59 (53)	8 (40)
Accumulated succes after 2nd ESWL	202 (84)	13 (76)	128 (88)	26 (79)	278 (89)	25 (86)	85 (77)	14 (70)
Accumulated succes after ≥ 3rd ESWL	223 (93)	15 (88)	136 (93)	27 (82)	296 (95)	26 (90)	99 (89)	16 (80)

## Discussion

The overall rate of stone clearance in this study was found to be in line with the reported stone free rate of centres with static machines. The overall stone free rate of 93% found in this study is significantly different from what was described in a recent study by Nafie et al., reporting a stone clearance rate of 49% [7]. In the study by Nafie it was speculated whether the low rate of stone clearance was due to a relative high proportion of patient with stones in the lower pole (43.3%). This is in significant contrast to a stone clearance of 93% for lower pole stones found in the present study, were 44% of the stones were located in the lower pole.

With regard to patient selection, information on the lower pole anatomy was not available and might have favoured stone clearance rates in this study but is unlikely to account for such large differences. Furthermore, the efficacy of ESWL is very dependent on the skills of the technician performing the treatment and in the present study treatments were carried out by a small team of very skilled and dedicated technicians [8]. In a previous multicentre study it was demonstrated that a transportable ESWL system had a high margin of safety with low complications rates and no apparent sacrifice of efficacy with regard to non-transportable systems [9].

Management of stones in the lower calyx using ESWL remains somewhat controversial. It has been reported that lower pole stones carry a lower success rate after ESWL monotherapy compared to stones in upper and middle calyx [1, 10]. However, a study with 246 cases of lower pole stones treated with EWSL concluded that stone size rather than lower pole anatomy was predictive of the efficacy [11]. Another study with nearly 600 renal stones fund no significant difference in stone clearance rate between stones located in the lower, middle and upper pole [12]. In the present study, no significant difference in ESWL success rate was observed between intra renal stone locations. Stones in the lower calyx were just as sensitive to ESWL as stones in other intra renal locations.

With a retreatment rate of 31%, the majority of stones required only a single treatment. However, the slightly higher retreatment rate found in this study compared to other series is consistent with the department’s liberal policy towards ESWL. By performing a second treatment of the stones that was initially unsuccessfully treated, the overall stone free rate increased from 69% to 87%. Performing three or more ESWL attempts only increases the overall success rate slightly from 87% to 93%. Based on the finding in the present study, it seems reasonable to offer a patient who was initially unsuccessful treated with ESWL at least one other attempt before considering invasive procedures.

The literature contains only little information on JJ-usage in relation to ESWL treatments and indications remains unclear and without consensus. The intention of a JJ is to prevent complications associated with ureteral obstruction as stone fragments is cleared trough the ureter. Conversely, the main drawbacks of JJ are bladder and kidney discomfort, risk of infections and calcification of the JJ. A survey among American urologists reported the JJ usage prior to ESWL to be 28% for 10 mm stones, 57% for 15 mm stones and 87% for 20 mm stones [13]. The results in the present study indicate a similar correlation between stone size and treatment with JJ prior to ESWL, though the results did not reach statistically significance. With respect to intra renal stone location, we found that significantly more patients with stones in the renal pelvis than elsewhere in the kidney were treated with JJ prior to ESWL. As expected, stones in the renal pelvis did give rise to to JJ-demanding obstruction more frequently than other intra renal locations. Previously, only a few studies have reported on JJ usage in relation to ESWL and concludes that treatment with a JJ prior to ESWL does not significantly influence stone free rates but generally results in more discomfort [14–17]. In the group of patients not treated with JJ prior to ESWL, we found that only 17 patients (3%) subsequently required treatment with an acute JJ because of complications associated with ureteral obstruction. Furthermore, we found association between stone size or intra renal stone location and the risk of needing treatment with JJ after the ESWL procedure.

The present study highlights the discrepancy in the efficacy of ESWL being reported in the literature, especially regarding mobile lithotripsy service. Although this study presents a large number of stones treated with EWSL the study is limited to being a single-centre design. Also, the retrospective design raises the issue of potential selection bias which are likely to have influenced the results of the present study. Further studies are warranted into the efficacy of mobile lithotripsy service, learning curve for ESWL technicians and commercialised health care services in general.

## Conclusions

The initial success rate after one ESWL procedure was 69%, which increased to an overall success rate of 93% after repeated treatment. Apart from the upper calyx, intra renal stone location was not associated with treatment efficacy, whereas an inverse relation was found between stone size and treatment efficacy. Patients that did not require treatment with a JJ prior to ESWL had only a minimal risk of needing such treatment subsequently, thus prophylactic placement before or after ESWL cannot be recommended.

## Abbreviations

CT: Computed tomography
EAU: European Association of Urology
ESWL: Extracorporeal shock wave lithotripsy
JJ: Internal ureteral double-J stent
PNL: Percutaneous nephrolithotomy
RIRS: Retrograde intrarenal surgery
